# Getting Better Acquainted with Auditory Voice Hallucinations (AVHs): A Need for Clinical and Social Change

**DOI:** 10.3389/fpsyg.2017.01978

**Published:** 2017-11-14

**Authors:** Antonio Iudici, Elena Faccio, Maria Quarato, Jessica Neri, Gianluca Castelnuovo

**Affiliations:** ^1^Department of Philosophy, Sociology, Education and Applied Psychology, University of Padova, Padua, Italy; ^2^Interactionist School of Padova, Istituto di Psicologia e Psicoterapia, Padua, Italy; ^3^Department of Psychology, Catholic University of the Sacred Heart, Milan, Italy; ^4^Istituto Auxologico Italiano IRCCS, Psychology Research Laboratory, Ospedale San Giuseppe, Verbania, Italy

**Keywords:** auditory verbal hallucination, psychopathology, clinical psychology, social sciences, psychiatry, hallucination experiences, clinical competence

## Introduction

The phenomenon of hearing voices (AVHs) is very much a subject of current scientific interest, both clinically^1^ and socially. For a long time, auditory hallucinations—perceiving sounds without external stimuli (David, 2004)—were considered an obvious sign of schizophrenic or psychotic psychopathology (Goodwin et al., 1971; Larøi et al., 2012), but these days such an association is no longer taken for granted. Various recent studies in the areas of psychology, psychiatry, and neuroscience have brought a renewal of interest in AVHs. First of all, the move beyond Kraepelinian logic (van Os, 2009; Fusar-Poli et al., 2014) has led us to see AVHs as a phenomenon in their own right, and not just a characteristic of schizophrenia (Fernyhough, 2004). Furthermore, a number of studies in imaging techniques have allowed us to study the phenomenon live, as it occurs, collecting various new data (Shergill et al., 2000). On the other hand, psychological studies with attempts at modeling, have boosted the idea that AVHs are linked to the linguistic and verbal qualities of the subject, thus reducing the association between voice hallucinations and signs of pathology (Johns and van Os, 2001; Pearson et al., 2001; Stanghellini and Cutting, 2003).

Other researchers have theorized that hearing voices is a different manifestation of self-awareness (Salvini and Bottini, 2011; Salvini and Quarato, 2011).

Even DSM-5 has modified the importance it attaches to hallucinations, in fact although the 4th edition diagnosed “schizophrenia” simply on the basis of the symptom “hallucinations,” in the new edition hallucinations on their own are not considered a sufficient symptom to diagnose the specter of schizophrenia” (American Psychiatric Association, 2013). Many of those suffering from this condition are not under treatment and are not diagnosable in psychopathological terms, which asks ever more questions of health professionals (Iudici, 2015), and which brings with it the risk that the phenomenon of hearing voices may be considered pathological because of a lack of understanding of the problem.

One direct implication of this risk concerns non-psychotic and non-schizophrenic hearers of voices who are afraid of being considered mad or disturbed, who very often live in fear for years without talking about it with anyone, although realizing that hearing voices causes no general maladjustment in their lives (Andrew et al., 2008). In the long term this can lead to feelings of alarm in some of them, and when such situations result in a visit to a clinic or a psychiatrist, there are often “suffering and conflicted confessions” about such experiences, especially by people who have never had psychiatric experience (Iudici and Gagliardo Corsi, 2017). These people consequently do not have appropriate information to help them understand their experiences (Faccio et al., 2013). This fact raises further doubts about the direct juxtaposition of auditory hallucinations and diagnoses of mental disturbance, and consequently our interest is in sensitizing clinicians to a broader interpretation of the phenomenon than the traditional view, highlighting the importance of considering more perspectives.

## Understanding AVHs: new research perspectives

There are various approaches which have recently offered a contribution to the understanding and management of AVHs and, in addition to the traditional psychological methods, we would point to psychological and phenomenological perspectives, cognitive neurobiology, and neuroimaging.

Psychological approaches presuppose that there is some continuity between AVHs and normal experience, and focus attention on the vocal content and on how it is interpreted and managed by people (van Os et al., 2000). The voices heard may take the form of a dialogue, may often be impersonated, or have a specific meaning for the individuals who experience them (David, 2004). They can be associated with psychological suffering, but may be a positive experience, for example when voices serve as a guide and reduce social isolation (Miller et al., 1993; Honig et al., 1998). Romme and Escher (1989) carried out research on 450 voice hearers found by the viewers of a popular German TV show, and a third of these appeared to be sane and have a positive relationship (coping) with their hallucinations. In research into hallucinatory experiences, Bentall (2000, p. 95) concluded that “the finding that a substantial minority of the population experiences frank hallucinations at some point in their lives must be considered very robust.” Many writers have observed that AVHs, in the absence of a diagnosis of mental disturbance, do not interfere with the activities of everyday life (Lawrence et al., 2010; Daalman et al., 2011; Badcock and Chhabra, 2013; Hill and Linden, 2013).

According to Liester (1996), psychotic and non-psychotic hallucinations are perceived in the same way, whereas they actually differ according to the type of personal experience that results from them, as also confirmed by Chadwick and Birchwood (1994). In particular, perceptions and beliefs regarding identity (hearing the voice of someone else distinct from oneself) and the interpersonal disposition (intentionality) toward the voices may appear to be highly relevant aspects (Mawson et al., 2010).

Saavedra et al. (2012) utilize Harre and van Langenhov's (1999) theories on the building of social interactions to understand the relationship between the voices and the subjects who hear them. In particular, the authors consider the characteristics of moral positioning, the conversational story and conversational ability in redefining interactions, within which hallucinatory experiences can be generated. Moreover, we should stress the practical characteristics connected with hearing AVHs as spoken phenomena (Leudar and Thomas, 2000).

Other research (Stanghellini and Cutting, 2003; Seal et al., 2004) has highlighted how it is possible to talk to ourselves in subvocal language. This research considers the possibility that in the formation of certain auditory hallucinations there is an inner subvocal language which subjects see as separate from themselves. The subvocalizations, made possible by the activation of the muscles regulating the articulation of words, occur during normal verbal thought. This activity may be accentuated when voices are heard (Faccio et al., 2016).

Fernyhough (2004) developed a descriptive model of AVHs. He proposed that inner speech, as a product of ontogenetic development, retains the dialogical qualities of socially mediated exchanges. Thus, when a dialogue is internalized, the different perspectives of the reality that are manifested in this dialogue are also internalized. The key to understanding the differences between these two forms of inner speech is in the transformations that accompany the internalization. The voices could be caused by a difficulty in the internalization of the socially mediated outer dialogue (Iudici and Verdecchia, 2015).

Then there is a tradition of phenomenological studies attempting to observe how hearers of voices live with their experience (Longden et al., 2012; McCarthy-Jones et al., 2013), which means getting involved with the subjects themselves, with whom it is possible to describe a journey of understanding the voices. Nayani and David (1996) carried out a phenomenological study on AVHs in patients diagnosed with schizophrenia, observing a model of AVHs increasing in complexity over time, with the addition of extra voices, extended dialogues, and greater intimacy between subjects and voices.

Over the years, neuropsychological studies have defined a number of models to explain AVHs, including misattribution of inner speech, language production, aberrant memory, and early automatic sensory processing errors. Studies of the misattribution of inner speech maintain that the patients have poor monitoring of how thoughts are self-generated because of compromised cognition, meaning that they attribute the thoughts to outside agents (Frith and Done, 1998). Others maintain that this process is due to a neuronal corollary discharge which does not allow the person to recognize their own thoughts (McGuire et al., 1995; de Weijer et al., 2013). Smith et al. (2013) have examined the relationship between linguistic production, voice production and perception, appreciating especially that AVHs are associated with cortical areas linked to linguistic functions. Such studies have in fact opened up the need to examine the functional aspects of this topic rather than the anatomical ones. In the aberrant memory model, the AVHs are due to a failure of recall inhibition which produces an involuntary activation of the memory, which provokes intrusive memories which emerge “out of context”, and a final perception of “otherness” (Jardri et al., 2011). Then, there is the model of early automatic sensory processing errors, in which the AVHs would be activated following impaired auditory processing and pre-attentive operation, which would lead to incorrect interpretation of the voices (Rosenfeld et al., 2011).

As for neuroimaging studies, looking at matters structurally some writers have noted a correlation between the presence of AVHs and certain structural changes in the gray matter volume (GMV) (Upthegrove et al., 2015). Some authors have found white matter (WM) deficits in the frontal and temporal areas associated with AVHs, suggesting that disconnection in the left frontotemporal area can contribute to the physiopathology of AVHs in schizophrenia (Seok et al., 2007). A lower connectivity in the left frontotemporal areas can be associated with AVHs as crucial areas implied in the auditory information processing. The mechanism underlying this functional disconnectivity involves significant WM changes in those regions consisting of a long association fiber tract. Furthermore, to evaluate the integrity of fiber tracts and myelination of neural axons in the WM, a specific index is used, that is the FA (fractional anisotropy) and a reduction of FA, in the frontal part of one of these fiber tracts (SLF) was detected, as well as increases of WM density in the temporal part of one another of these fiber tracts (ILF).

Looking at matters functionally, functional MRI scans (fMRI) of patients with schizophrenia and AVHs have discovered that when the brain is in a resting state—not occupied with direct activity—there is reduced connectivity in the primary and secondary auditory cortex and in the areas of linguistic production (Mechelli et al., 2007). Based on evidence of a resting state, various fMRI studies have recently highlighted raised activity in the default mode network (DMN^2^) (Vercammen et al., 2010). Mechelli et al. (2007) and Vercammen et al. (2010) highlight different aspects related to the phenomenology and functional dimension of AVHs. They focus attention on the process of appraisal and (mis) attribution to an external source rather than an inner thinking or a patient's own speech with changes and abnormal patterns in the particular regions described. Although, the neuropsychological models and neuroimaging studies have led to a more precise evaluation of AVHs, so far no model seems to exhaust the great complexity of the phenomena found in the various studies (Upthegrove et al., 2016). It does, however, seem increasingly clear that what is needed is a dimensional and functional approach rather than one based on categories (Iudici et al., 2015), showing AVHs in terms of a variable experiential continuum, and not necessarily pathological.

## Conclusions and operational implications

In the last 20 years the subject of AVHs has aroused lively interest in the scientific community. Apart from the nosographic approach, other perspectives—psychological, phenomenological, and neuropsychological—are contributing to a clearer understanding of the experience of hearing voices. AVHs, with the exception of certain medical conditions, can increasingly be seen as a normal experience for human beings, which takes different forms through subjective and interactive language-building processes (Saavedra et al., 2012). This is a phenomenon which interests and involves large numbers of people, and its sound management is a social necessity. It involves all those people, whether patients or not, successful professionals or ordinary people, who hear voices and, seeing them as a psychiatric symptom, do not talk to anyone about them, fearing that they will be judged. It should be added, though, that according to some studies many of these people are able to deal with them effectively, turning them into an enriching experience (Dillon and Hornstein, 2013; McCarthy-Jones and Longden, 2013).

In the field of mental health, for the healthcare professionals who may come into contact with patients who hear voices, what we have discussed here may turn out to be of vital importance, both to arrive at a better understanding of AVHs and to become proficient at managing them.

## Author contributions

AI has been involved in the Study Conception and Design. EF, JN, and MQ have been involved in data acquisition. EF, AI, and GC have been involved in the analysis and interpretation of data and the drafting of manuscripts. GC and AI have been critical of the critical revision.

### Conflict of interest statement

The authors declare that the research was conducted in the absence of any commercial or financial relationships that could be construed as a potential conflict of interest.

## Footnotes

^1^The phenomenon seems to affect about 13.2% of the general population (Johns et al., 2014).

^2^The DMN is a network of brain regions which are active when the individual is not concentrating on the outside world and the brain is at wakeful rest (Whitfield-Gabrieli and Ford, 2012).
